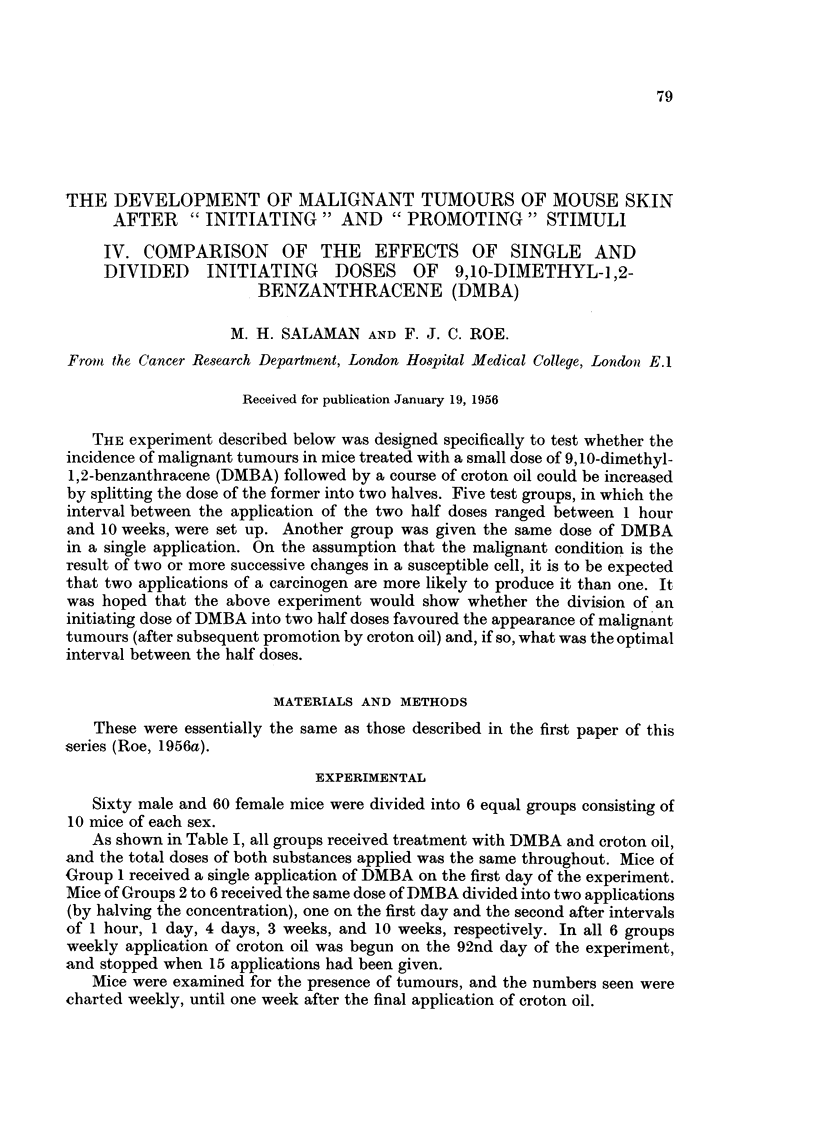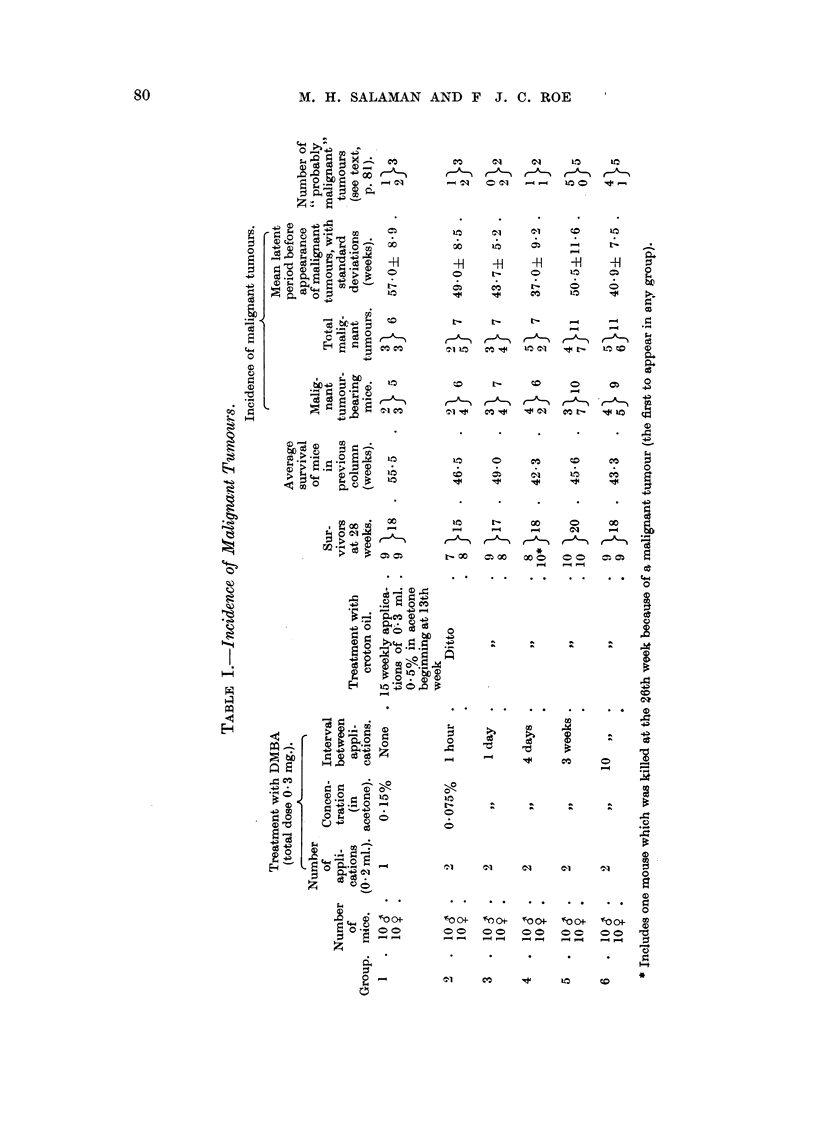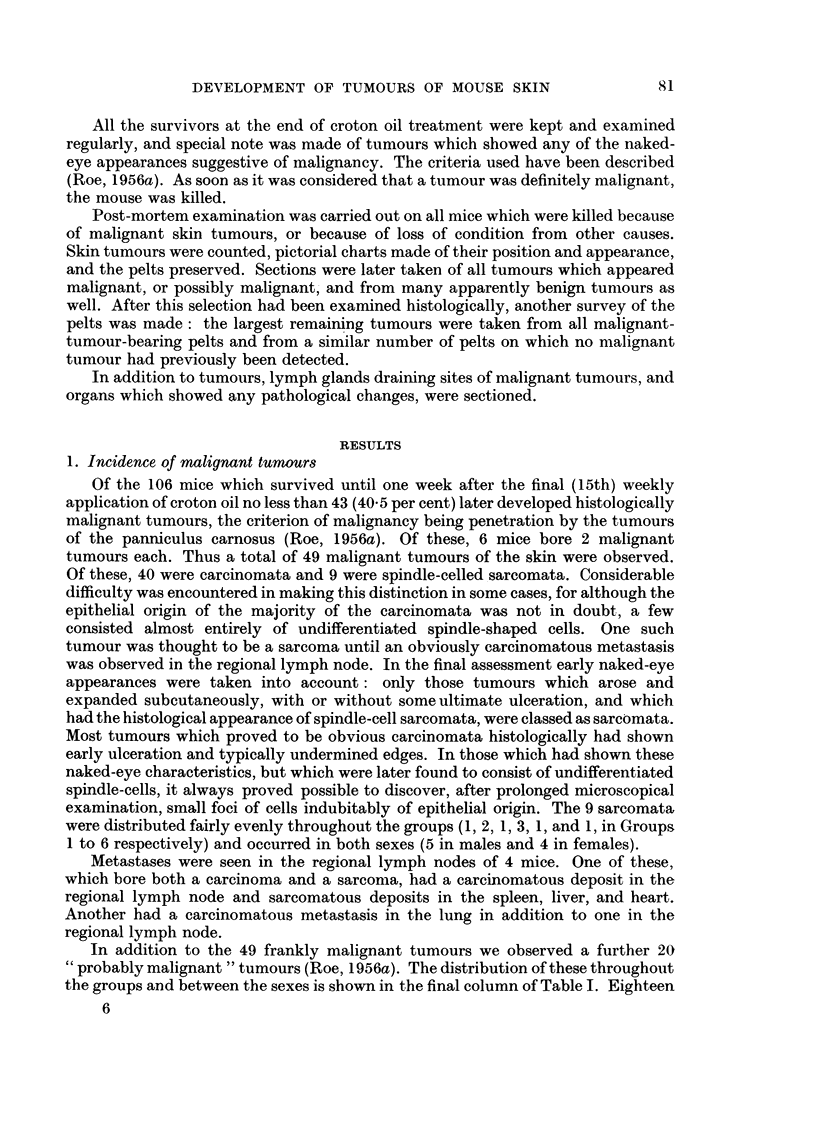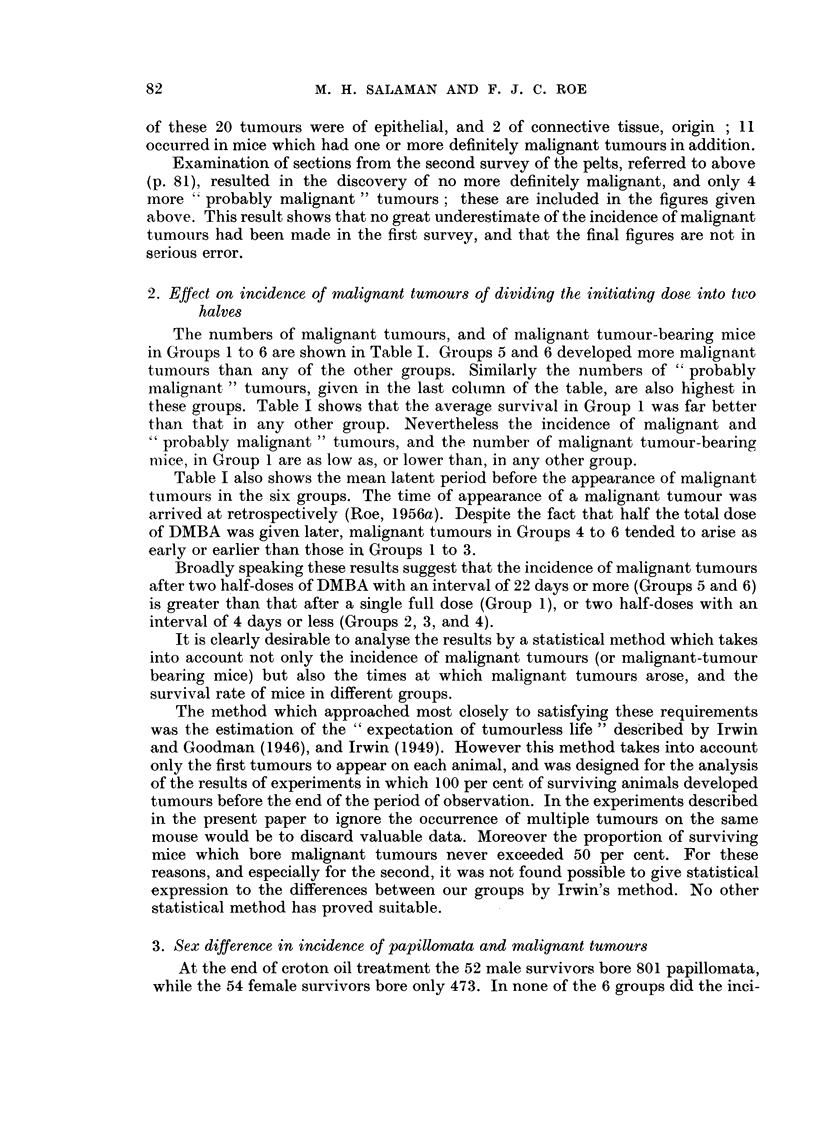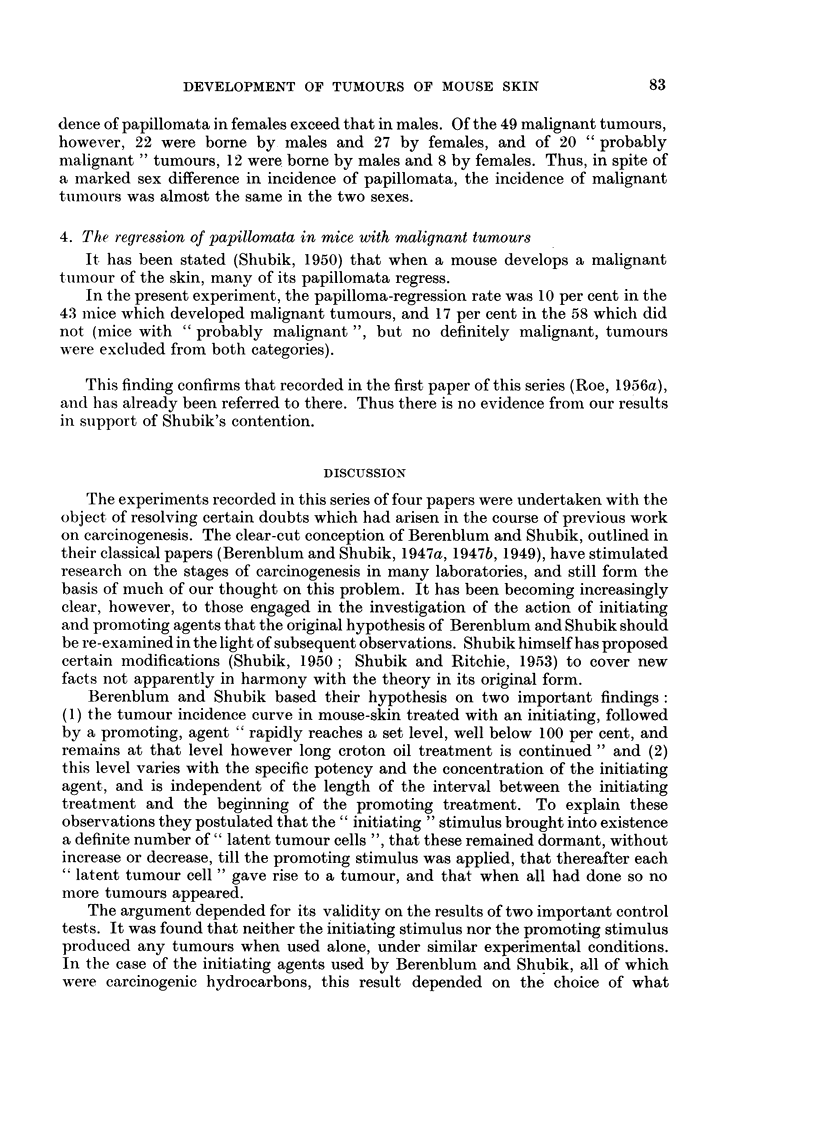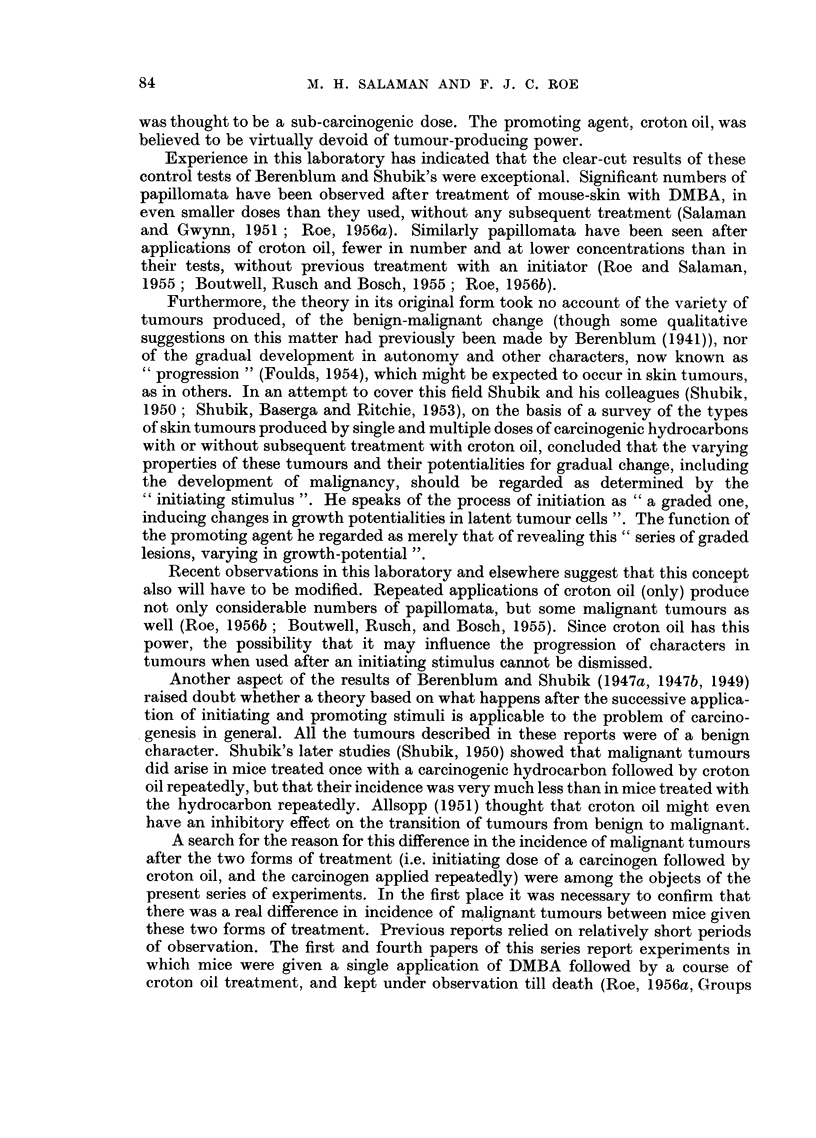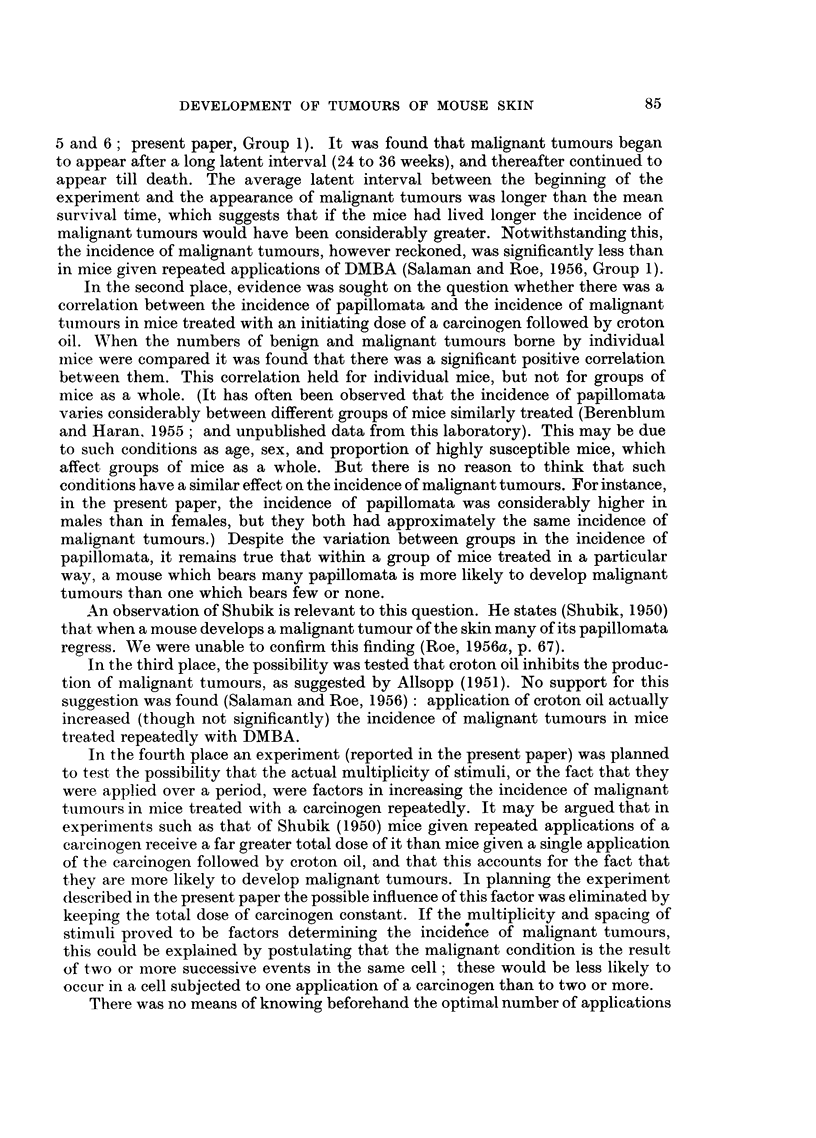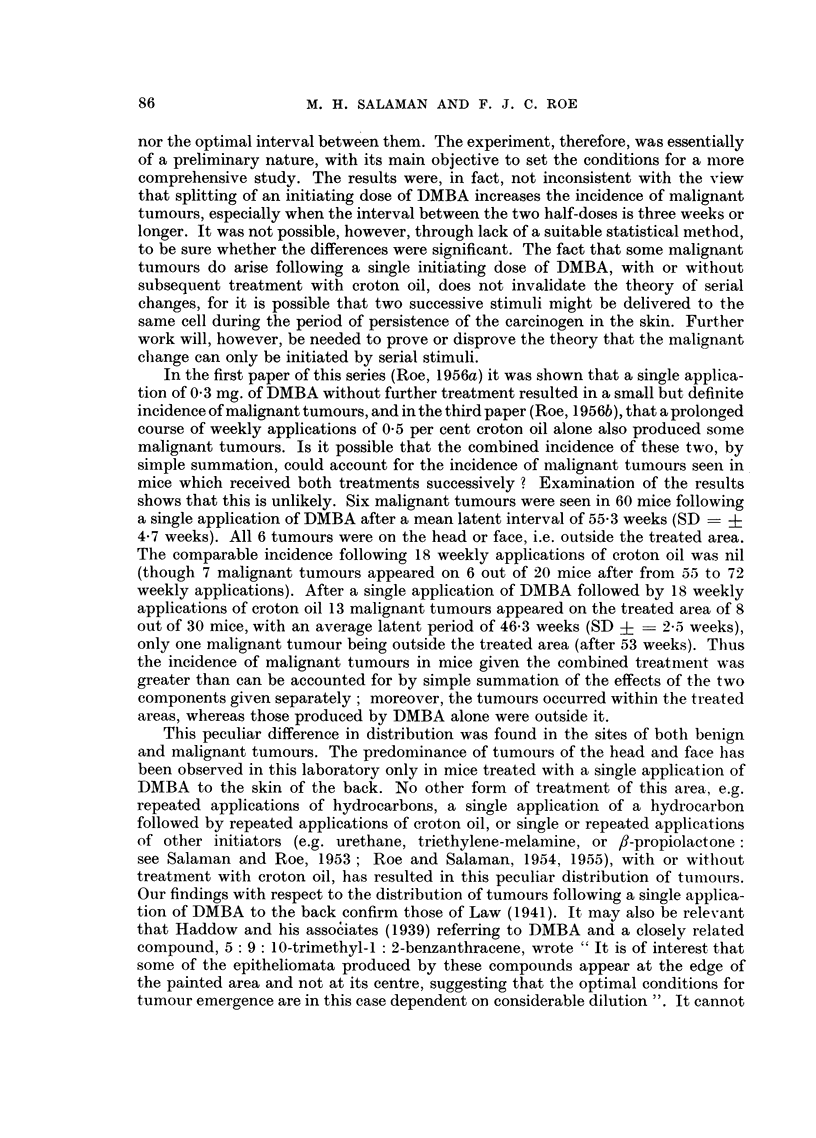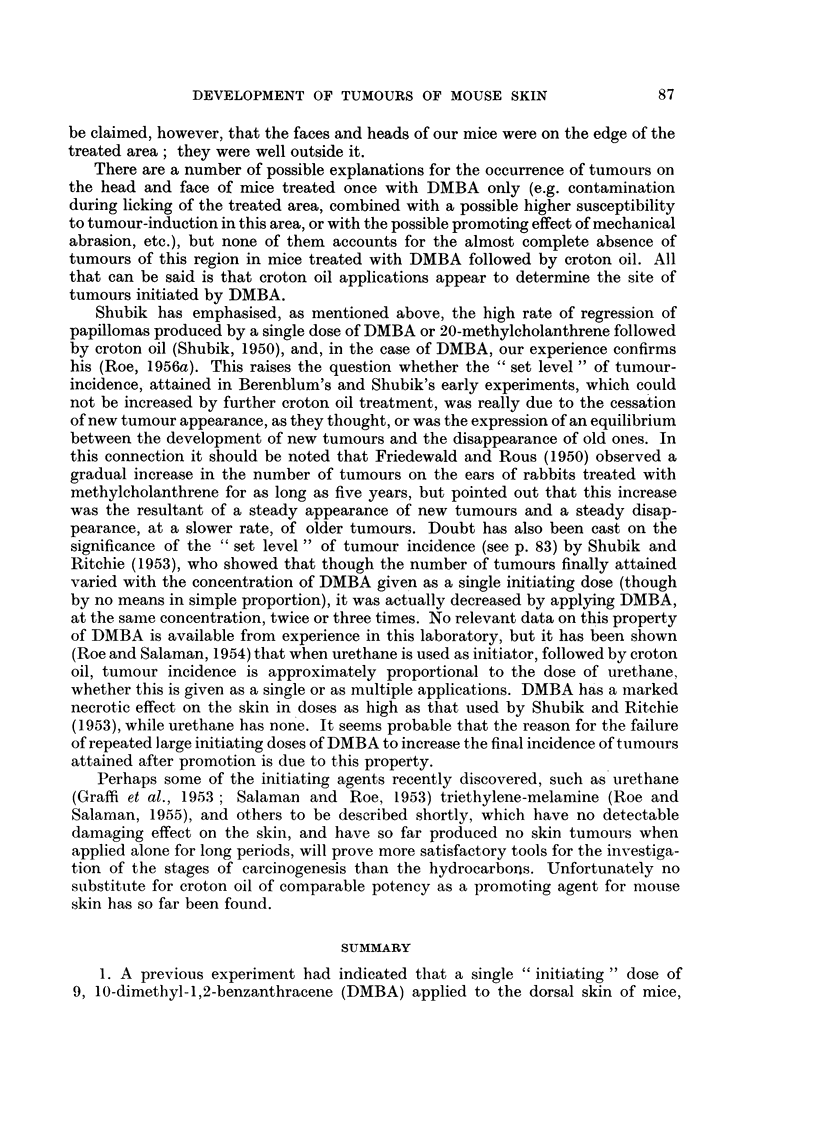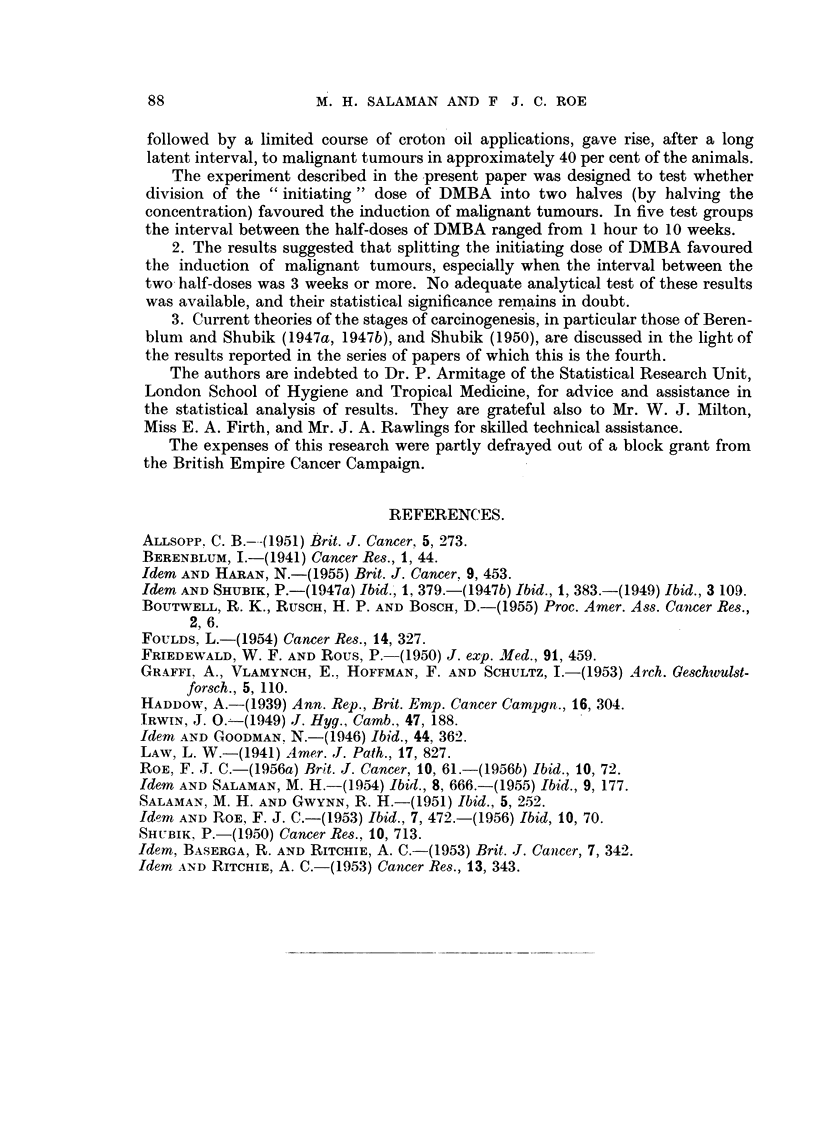# The Development of Malignant Tumours of Mouse Skin after “Initiating” and “Promoting” Stimuli. IV. Comparison of the Effects of Single and Divided Initiating Doses of 9, 10-Dimethyl-1, 2-Benzanthrene (DMBA)

**DOI:** 10.1038/bjc.1956.12

**Published:** 1956-03

**Authors:** M. H. Salaman, F. J. C. Roe


### THE DEVELOPMENT OF MALIGNANT TUMOURS OF MOUSE SKIN AFTER " INITIATING " AND " PROMOTING " STIMULI

### IV. COMPARISON OF THE EFFECTS OF SINGLE AND DIVIDED INITIATING DOSES OF 9,10-DIMETHYL-1,2-BENZANTHRACENE (DMBA)

M. H. SALAMAN AND F. J. C. ROE.

*From the Cancer Research Department, London Hospital Medical College, London E.*1

Received for publication January 19, 1956

THE experiment described below was designed specifically to test whether the incidence of malignant tumours in mice treated with a small dose of 9,10-dimethyl-1,2-benzanthracene (DMBA) followed by a course of croton oil could be increased by splitting the dose of the former into two halves. Five test groups, in which the interval between the application of the two half doses ranged between 1 hour and 10 weeks, were set up. Another group was given the same dose of DMBA in a single application. On the assumption that the malignant condition is the result of two or more successive changes in a susceptible cell, it is to be expected that two applications of a carcinogen are more likely to produce it than one. It was hoped that the above experiment would show whether the division of an initiating dose of DMBA into two half doses favoured the appearance of malignant tumours (after subsequent promotion by croton oil) and, if so, what was the optimal interval between the half doses.

### MATERIALS AND METHODS

These were essentially the same as those described in the first paper of this series (Roe, 1956*a*).

### EXPERIMENTAL

Sixty male and 60 female mice were divided into 6 equal groups consisting of 10 mice of each sex.

As shown in Table I, all groups received treatment with DMBA and croton oil, and the total doses of both substances applied was the same throughout. Mice of Group 1 received a single application of DMBA on the first day of the experiment. Mice of Groups 2 to 6 received the same dose of DMBA divided into two applications (by halving the concentration), one on the first day and the second after intervals of 1 hour, 1 day, 4 days, 3 weeks, and 10 weeks, respectively. In all 6 groups weekly application of croton oil was begun on the 92nd day of the experiment, and stopped when 15 applications had been given.

Mice were examined for the presence of tumours, and the numbers seen were charted weekly, until one week after the final application of croton oil.

TABLE I.—*Incidence of Malignant Tumours.*

| Group. | Number of mice. | Treatment with DMBA (total dose 0·3 mg.). Number of applications (0·2 ml.). | Concentration (in acetone). | Interval between applications. | Treatment with croton oil. | Survivors at 28 weeks. | Average survival of mice in previous column (weeks). | Incidence of malignant tumours. Malignant tumour-bearing mice. | Total malignant tumours. | Mean latent period before appearance of malignant tumours, with standard deviations (weeks). | Number of "probably malignant" tumours (see text, p. 81). |
|---|---|---|---|---|---|---|---|---|---|---|---|
| 1 | 10 ♂<br>10 ♀ | 1 | 0·15% | None | 15 weekly applications of 0·3 ml. 0·5% in acetone beginning at 13th week | 9<br>9 } 18 | 55·5 | 2<br>3 } 5 | 3<br>3 } 6 | 57·0± 8·9 | 1<br>2 } 3 |
| 2 | 10 ♂<br>10 ♀ | 2 | 0·075% | 1 hour | Ditto | 7<br>8 } 15 | 46·5 | 2<br>4 } 6 | 2<br>5 } 7 | 49·0± 8·5 | 1<br>2 } 3 |
| 3 | 10 ♂<br>10 ♀ | 2 | " | 1 day | " | 9<br>8 } 17 | 49·0 | 3<br>4 } 7 | 3<br>4 } 7 | 43·7± 5·2 | 0<br>2 } 2 |
| 4 | 10 ♂<br>10 ♀ | 2 | " | 4 days | " | 8<br>10* } 18 | 42·3 | 4<br>2 } 6 | 5<br>2 } 7 | 37·0± 9·2 | 1<br>1 } 2 |
| 5 | 10 ♂<br>10 ♀ | 2 | " | 3 weeks | " | 10<br>10 } 20 | 45·6 | 3<br>7 } 10 | 4<br>7 } 11 | 50·5±11·6 | 5<br>0 } 5 |
| 6 | 10 ♂<br>10 ♀ | 2 | " | 10 " | " | 9<br>9 } 18 | 43·3 | 4<br>5 } 9 | 5<br>6 } 11 | 40·9± 7·5 | 4<br>1 } 5 |

* Includes one mouse which was killed at the 26th week because of a malignant tumour (the first to appear in any group).

All the survivors at the end of croton oil treatment were kept and examined regularly, and special note was made of tumours which showed any of the naked-eye appearances suggestive of malignancy. The criteria used have been described (Roe, 1956*a*). As soon as it was considered that a tumour was definitely malignant, the mouse was killed.

Post-mortem examination was carried out on all mice which were killed because of malignant skin tumours, or because of loss of condition from other causes. Skin tumours were counted, pictorial charts made of their position and appearance, and the pelts preserved. Sections were later taken of all tumours which appeared malignant, or possibly malignant, and from many apparently benign tumours as well. After this selection had been examined histologically, another survey of the pelts was made : the largest remaining tumours were taken from all malignant-tumour-bearing pelts and from a similar number of pelts on which no malignant tumour had previously been detected.

In addition to tumours, lymph glands draining sites of malignant tumours, and organs which showed any pathological changes, were sectioned.

### RESULTS

### 1. *Incidence of malignant tumours*

Of the 106 mice which survived until one week after the final (15th) weekly application of croton oil no less than 43 (40·5 per cent) later developed histologically malignant tumours, the criterion of malignancy being penetration by the tumours of the panniculus carnosus (Roe, 1956*a*). Of these, 6 mice bore 2 malignant tumours each. Thus a total of 49 malignant tumours of the skin were observed. Of these, 40 were carcinomata and 9 were spindle-celled sarcomata. Considerable difficulty was encountered in making this distinction in some cases, for although the epithelial origin of the majority of the carcinomata was not in doubt, a few consisted almost entirely of undifferentiated spindle-shaped cells. One such tumour was thought to be a sarcoma until an obviously carcinomatous metastasis was observed in the regional lymph node. In the final assessment early naked-eye appearances were taken into account : only those tumours which arose and expanded subcutaneously, with or without some ultimate ulceration, and which had the histological appearance of spindle-cell sarcomata, were classed as sarcomata. Most tumours which proved to be obvious carcinomata histologically had shown early ulceration and typically undermined edges. In those which had shown these naked-eye characteristics, but which were later found to consist of undifferentiated spindle-cells, it always proved possible to discover, after prolonged microscopical examination, small foci of cells indubitably of epithelial origin. The 9 sarcomata were distributed fairly evenly throughout the groups (1, 2, 1, 3, 1, and 1, in Groups 1 to 6 respectively) and occurred in both sexes (5 in males and 4 in females).

Metastases were seen in the regional lymph nodes of 4 mice. One of these, which bore both a carcinoma and a sarcoma, had a carcinomatous deposit in the regional lymph node and sarcomatous deposits in the spleen, liver, and heart. Another had a carcinomatous metastasis in the lung in addition to one in the regional lymph node.

In addition to the 49 frankly malignant tumours we observed a further 20 " probably malignant " tumours (Roe, 1956*a*). The distribution of these throughout the groups and between the sexes is shown in the final column of Table I. Eighteen of these 20 tumours were of epithelial, and 2 of connective tissue, origin ; 11 occurred in mice which had one or more definitely malignant tumours in addition.

Examination of sections from the second survey of the pelts, referred to above (p. 81), resulted in the discovery of no more definitely malignant, and only 4 more " probably malignant " tumours ; these are included in the figures given above. This result shows that no great underestimate of the incidence of malignant tumours had been made in the first survey, and that the final figures are not in serious error.

2. *Effect on incidence of malignant tumours of dividing the initiating dose into two halves*

The numbers of malignant tumours, and of malignant tumour-bearing mice in Groups 1 to 6 are shown in Table I. Groups 5 and 6 developed more malignant tumours than any of the other groups. Similarly the numbers of " probably malignant " tumours, given in the last column of the table, are also highest in these groups. Table I shows that the average survival in Group 1 was far better than that in any other group. Nevertheless the incidence of malignant and " probably malignant " tumours, and the number of malignant tumour-bearing mice, in Group 1 are as low as, or lower than, in any other group.

Table I also shows the mean latent period before the appearance of malignant tumours in the six groups. The time of appearance of a malignant tumour was arrived at retrospectively (Roe, 1956*a*). Despite the fact that half the total dose of DMBA was given later, malignant tumours in Groups 4 to 6 tended to arise as early or earlier than those in Groups 1 to 3.

Broadly speaking these results suggest that the incidence of malignant tumours after two half-doses of DMBA with an interval of 22 days or more (Groups 5 and 6) is greater than that after a single full dose (Group 1), or two half-doses with an interval of 4 days or less (Groups 2, 3, and 4).

It is clearly desirable to analyse the results by a statistical method which takes into account not only the incidence of malignant tumours (or malignant-tumour bearing mice) but also the times at which malignant tumours arose, and the survival rate of mice in different groups.

The method which approached most closely to satisfying these requirements was the estimation of the " expectation of tumourless life " described by Irwin and Goodman (1946), and Irwin (1949). However this method takes into account only the first tumours to appear on each animal, and was designed for the analysis of the results of experiments in which 100 per cent of surviving animals developed tumours before the end of the period of observation. In the experiments described in the present paper to ignore the occurrence of multiple tumours on the same mouse would be to discard valuable data. Moreover the proportion of surviving mice which bore malignant tumours never exceeded 50 per cent. For these reasons, and especially for the second, it was not found possible to give statistical expression to the differences between our groups by Irwin's method. No other statistical method has proved suitable.

3. *Sex difference in incidence of papillomata and malignant tumours*

At the end of croton oil treatment the 52 male survivors bore 801 papillomata, while the 54 female survivors bore only 473. In none of the 6 groups did the incidence of papillomata in females exceed that in males. Of the 49 malignant tumours, however, 22 were borne by males and 27 by females, and of 20 " probably malignant " tumours, 12 were borne by males and 8 by females. Thus, in spite of a marked sex difference in incidence of papillomata, the incidence of malignant tumours was almost the same in the two sexes.

4. *The regression of papillomata in mice with malignant tumours*

It has been stated (Shubik, 1950) that when a mouse develops a malignant tumour of the skin, many of its papillomata regress.

In the present experiment, the papilloma-regression rate was 10 per cent in the 43 mice which developed malignant tumours, and 17 per cent in the 58 which did not (mice with " probably malignant ", but no definitely malignant, tumours were excluded from both categories).

This finding confirms that recorded in the first paper of this series (Roe, 1956*a*), and has already been referred to there. Thus there is no evidence from our results in support of Shubik's contention.

### DISCUSSION

The experiments recorded in this series of four papers were undertaken with the object of resolving certain doubts which had arisen in the course of previous work on carcinogenesis. The clear-cut conception of Berenblum and Shubik, outlined in their classical papers (Berenblum and Shubik, 1947*a*, 1947*b*, 1949), have stimulated research on the stages of carcinogenesis in many laboratories, and still form the basis of much of our thought on this problem. It has been becoming increasingly clear, however, to those engaged in the investigation of the action of initiating and promoting agents that the original hypothesis of Berenblum and Shubik should be re-examined in the light of subsequent observations. Shubik himself has proposed certain modifications (Shubik, 1950 ; Shubik and Ritchie, 1953) to cover new facts not apparently in harmony with the theory in its original form.

Berenblum and Shubik based their hypothesis on two important findings : (1) the tumour incidence curve in mouse-skin treated with an initiating, followed by a promoting, agent " rapidly reaches a set level, well below 100 per cent, and remains at that level however long croton oil treatment is continued " and (2) this level varies with the specific potency and the concentration of the initiating agent, and is independent of the length of the interval between the initiating treatment and the beginning of the promoting treatment. To explain these observations they postulated that the " initiating " stimulus brought into existence a definite number of " latent tumour cells ", that these remained dormant, without increase or decrease, till the promoting stimulus was applied, that thereafter each " latent tumour cell " gave rise to a tumour, and that when all had done so no more tumours appeared.

The argument depended for its validity on the results of two important control tests. It was found that neither the initiating stimulus nor the promoting stimulus produced any tumours when used alone, under similar experimental conditions. In the case of the initiating agents used by Berenblum and Shubik, all of which were carcinogenic hydrocarbons, this result depended on the choice of what was thought to be a sub-carcinogenic dose. The promoting agent, croton oil, was believed to be virtually devoid of tumour-producing power.

Experience in this laboratory has indicated that the clear-cut results of these control tests of Berenblum and Shubik's were exceptional. Significant numbers of papillomata have been observed after treatment of mouse-skin with DMBA, in even smaller doses than they used, without any subsequent treatment (Salaman and Gwynn, 1951 ; Roe, 1956*a*). Similarly papillomata have been seen after applications of croton oil, fewer in number and at lower concentrations than in their tests, without previous treatment with an initiator (Roe and Salaman, 1955 ; Boutwell, Rusch and Bosch, 1955 ; Roe, 1956*b*).

Furthermore, the theory in its original form took no account of the variety of tumours produced, of the benign-malignant change (though some qualitative suggestions on this matter had previously been made by Berenblum (1941)), nor of the gradual development in autonomy and other characters, now known as " progression " (Foulds, 1954), which might be expected to occur in skin tumours, as in others. In an attempt to cover this field Shubik and his colleagues (Shubik, 1950 ; Shubik, Baserga and Ritchie, 1953), on the basis of a survey of the types of skin tumours produced by single and multiple doses of carcinogenic hydrocarbons with or without subsequent treatment with croton oil, concluded that the varying properties of these tumours and their potentialities for gradual change, including the development of malignancy, should be regarded as determined by the " initiating stimulus ". He speaks of the process of initiation as " a graded one, inducing changes in growth potentialities in latent tumour cells ". The function of the promoting agent he regarded as merely that of revealing this " series of graded lesions, varying in growth-potential ".

Recent observations in this laboratory and elsewhere suggest that this concept also will have to be modified. Repeated applications of croton oil (only) produce not only considerable numbers of papillomata, but some malignant tumours as well (Roe, 1956*b* ; Boutwell, Rusch, and Bosch, 1955). Since croton oil has this power, the possibility that it may influence the progression of characters in tumours when used after an initiating stimulus cannot be dismissed.

Another aspect of the results of Berenblum and Shubik (1947*a*, 1947*b*, 1949) raised doubt whether a theory based on what happens after the successive application of initiating and promoting stimuli is applicable to the problem of carcinogenesis in general. All the tumours described in these reports were of a benign character. Shubik's later studies (Shubik, 1950) showed that malignant tumours did arise in mice treated once with a carcinogenic hydrocarbon followed by croton oil repeatedly, but that their incidence was very much less than in mice treated with the hydrocarbon repeatedly. Allsopp (1951) thought that croton oil might even have an inhibitory effect on the transition of tumours from benign to malignant.

A search for the reason for this difference in the incidence of malignant tumours after the two forms of treatment (i.e. initiating dose of a carcinogen followed by croton oil, and the carcinogen applied repeatedly) were among the objects of the present series of experiments. In the first place it was necessary to confirm that there was a real difference in incidence of malignant tumours between mice given these two forms of treatment. Previous reports relied on relatively short periods of observation. The first and fourth papers of this series report experiments in which mice were given a single application of DMBA followed by a course of croton oil treatment, and kept under observation till death (Roe, 1956*a*, Groups

5 and 6 ; present paper, Group 1). It was found that malignant tumours began to appear after a long latent interval (24 to 36 weeks), and thereafter continued to appear till death. The average latent interval between the beginning of the experiment and the appearance of malignant tumours was longer than the mean survival time, which suggests that if the mice had lived longer the incidence of malignant tumours would have been considerably greater. Notwithstanding this, the incidence of malignant tumours, however reckoned, was significantly less than in mice given repeated applications of DMBA (Salaman and Roe, 1956, Group 1).

In the second place, evidence was sought on the question whether there was a correlation between the incidence of papillomata and the incidence of malignant tumours in mice treated with an initiating dose of a carcinogen followed by croton oil. When the numbers of benign and malignant tumours borne by individual mice were compared it was found that there was a significant positive correlation between them. This correlation held for individual mice, but not for groups of mice as a whole. (It has often been observed that the incidence of papillomata varies considerably between different groups of mice similarly treated (Berenblum and Haran, 1955 ; and unpublished data from this laboratory). This may be due to such conditions as age, sex, and proportion of highly susceptible mice, which affect groups of mice as a whole. But there is no reason to think that such conditions have a similar effect on the incidence of malignant tumours. For instance, in the present paper, the incidence of papillomata was considerably higher in males than in females, but they both had approximately the same incidence of malignant tumours.) Despite the variation between groups in the incidence of papillomata, it remains true that within a group of mice treated in a particular way, a mouse which bears many papillomata is more likely to develop malignant tumours than one which bears few or none.

An observation of Shubik is relevant to this question. He states (Shubik, 1950) that when a mouse develops a malignant tumour of the skin many of its papillomata regress. We were unable to confirm this finding (Roe, 1956*a*, p. 67).

In the third place, the possibility was tested that croton oil inhibits the production of malignant tumours, as suggested by Allsopp (1951). No support for this suggestion was found (Salaman and Roe, 1956) : application of croton oil actually increased (though not significantly) the incidence of malignant tumours in mice treated repeatedly with DMBA.

In the fourth place an experiment (reported in the present paper) was planned to test the possibility that the actual multiplicity of stimuli, or the fact that they were applied over a period, were factors in increasing the incidence of malignant tumours in mice treated with a carcinogen repeatedly. It may be argued that in experiments such as that of Shubik (1950) mice given repeated applications of a carcinogen receive a far greater total dose of it than mice given a single application of the carcinogen followed by croton oil, and that this accounts for the fact that they are more likely to develop malignant tumours. In planning the experiment described in the present paper the possible influence of this factor was eliminated by keeping the total dose of carcinogen constant. If the multiplicity and spacing of stimuli proved to be factors determining the incidence of malignant tumours, this could be explained by postulating that the malignant condition is the result of two or more successive events in the same cell ; these would be less likely to occur in a cell subjected to one application of a carcinogen than to two or more.

There was no means of knowing beforehand the optimal number of applications nor the optimal interval between them. The experiment, therefore, was essentially of a preliminary nature, with its main objective to set the conditions for a more comprehensive study. The results were, in fact, not inconsistent with the view that splitting of an initiating dose of DMBA increases the incidence of malignant tumours, especially when the interval between the two half-doses is three weeks or longer. It was not possible, however, through lack of a suitable statistical method, to be sure whether the differences were significant. The fact that some malignant tumours do arise following a single initiating dose of DMBA, with or without subsequent treatment with croton oil, does not invalidate the theory of serial changes, for it is possible that two successive stimuli might be delivered to the same cell during the period of persistence of the carcinogen in the skin. Further work will, however, be needed to prove or disprove the theory that the malignant change can only be initiated by serial stimuli.

In the first paper of this series (Roe, 1956*a*) it was shown that a single application of 0·3 mg. of DMBA without further treatment resulted in a small but definite incidence of malignant tumours, and in the third paper (Roe, 1956*b*), that a prolonged course of weekly applications of 0·5 per cent croton oil alone also produced some malignant tumours. Is it possible that the combined incidence of these two, by simple summation, could account for the incidence of malignant tumours seen in mice which received both treatments successively ? Examination of the results shows that this is unlikely. Six malignant tumours were seen in 60 mice following a single application of DMBA after a mean latent interval of 55·3 weeks (SD = $\pm$ 4·7 weeks). All 6 tumours were on the head or face, i.e. outside the treated area. The comparable incidence following 18 weekly applications of croton oil was nil (though 7 malignant tumours appeared on 6 out of 20 mice after from 55 to 72 weekly applications). After a single application of DMBA followed by 18 weekly applications of croton oil 13 malignant tumours appeared on the treated area of 8 out of 30 mice, with an average latent period of 46·3 weeks (SD $\pm$ = 2·5 weeks), only one malignant tumour being outside the treated area (after 53 weeks). Thus the incidence of malignant tumours in mice given the combined treatment was greater than can be accounted for by simple summation of the effects of the two components given separately ; moreover, the tumours occurred within the treated areas, whereas those produced by DMBA alone were outside it.

This peculiar difference in distribution was found in the sites of both benign and malignant tumours. The predominance of tumours of the head and face has been observed in this laboratory only in mice treated with a single application of DMBA to the skin of the back. No other form of treatment of this area, e.g. repeated applications of hydrocarbons, a single application of a hydrocarbon followed by repeated applications of croton oil, or single or repeated applications of other initiators (e.g. urethane, triethylene-melamine, or $\beta$-propiolactone : see Salaman and Roe, 1953 ; Roe and Salaman, 1954, 1955), with or without treatment with croton oil, has resulted in this peculiar distribution of tumours. Our findings with respect to the distribution of tumours following a single application of DMBA to the back confirm those of Law (1941). It may also be relevant that Haddow and his associates (1939) referring to DMBA and a closely related compound, 5 : 9 : 10-trimethyl-1 : 2-benzanthracene, wrote " It is of interest that some of the epitheliomata produced by these compounds appear at the edge of the painted area and not at its centre, suggesting that the optimal conditions for tumour emergence are in this case dependent on considerable dilution ". It cannot be claimed, however, that the faces and heads of our mice were on the edge of the treated area ; they were well outside it.

There are a number of possible explanations for the occurrence of tumours on the head and face of mice treated once with DMBA only (e.g. contamination during licking of the treated area, combined with a possible higher susceptibility to tumour-induction in this area, or with the possible promoting effect of mechanical abrasion, etc.), but none of them accounts for the almost complete absence of tumours of this region in mice treated with DMBA followed by croton oil. All that can be said is that croton oil applications appear to determine the site of tumours initiated by DMBA.

Shubik has emphasised, as mentioned above, the high rate of regression of papillomas produced by a single dose of DMBA or 20-methylcholanthrene followed by croton oil (Shubik, 1950), and, in the case of DMBA, our experience confirms his (Roe, 1956*a*). This raises the question whether the " set level " of tumour-incidence, attained in Berenblum's and Shubik's early experiments, which could not be increased by further croton oil treatment, was really due to the cessation of new tumour appearance, as they thought, or was the expression of an equilibrium between the development of new tumours and the disappearance of old ones. In this connection it should be noted that Friedewald and Rous (1950) observed a gradual increase in the number of tumours on the ears of rabbits treated with methylcholanthrene for as long as five years, but pointed out that this increase was the resultant of a steady appearance of new tumours and a steady disappearance, at a slower rate, of older tumours. Doubt has also been cast on the significance of the " set level " of tumour incidence (see p. 83) by Shubik and Ritchie (1953), who showed that though the number of tumours finally attained varied with the concentration of DMBA given as a single initiating dose (though by no means in simple proportion), it was actually decreased by applying DMBA, at the same concentration, twice or three times. No relevant data on this property of DMBA is available from experience in this laboratory, but it has been shown (Roe and Salaman, 1954) that when urethane is used as initiator, followed by croton oil, tumour incidence is approximately proportional to the dose of urethane, whether this is given as a single or as multiple applications. DMBA has a marked necrotic effect on the skin in doses as high as that used by Shubik and Ritchie (1953), while urethane has none. It seems probable that the reason for the failure of repeated large initiating doses of DMBA to increase the final incidence of tumours attained after promotion is due to this property.

Perhaps some of the initiating agents recently discovered, such as urethane (Graffi *et al.*, 1953 ; Salaman and Roe, 1953) triethylene-melamine (Roe and Salaman, 1955), and others to be described shortly, which have no detectable damaging effect on the skin, and have so far produced no skin tumours when applied alone for long periods, will prove more satisfactory tools for the investigation of the stages of carcinogenesis than the hydrocarbons. Unfortunately no substitute for croton oil of comparable potency as a promoting agent for mouse skin has so far been found.

### SUMMARY

1. A previous experiment had indicated that a single " initiating " dose of 9, 10-dimethyl-1,2-benzanthracene (DMBA) applied to the dorsal skin of mice, followed by a limited course of croton oil applications, gave rise, after a long latent interval, to malignant tumours in approximately 40 per cent of the animals.

The experiment described in the present paper was designed to test whether division of the "initiating" dose of DMBA into two halves (by halving the concentration) favoured the induction of malignant tumours. In five test groups the interval between the half-doses of DMBA ranged from 1 hour to 10 weeks.

2. The results suggested that splitting the initiating dose of DMBA favoured the induction of malignant tumours, especially when the interval between the two half-doses was 3 weeks or more. No adequate analytical test of these results was available, and their statistical significance remains in doubt.

3. Current theories of the stages of carcinogenesis, in particular those of Berenblum and Shubik (1947*a*, 1947*b*), and Shubik (1950), are discussed in the light of the results reported in the series of papers of which this is the fourth.

The authors are indebted to Dr. P. Armitage of the Statistical Research Unit, London School of Hygiene and Tropical Medicine, for advice and assistance in the statistical analysis of results. They are grateful also to Mr. W. J. Milton, Miss E. A. Firth, and Mr. J. A. Rawlings for skilled technical assistance.

The expenses of this research were partly defrayed out of a block grant from the British Empire Cancer Campaign.

### REFERENCES.

ALLSOPP, C. B.—(1951) *Brit. J. Cancer*, **5**, 273.
BERENBLUM, I.—(1941) *Cancer Res.*, **1**, 44.
*Idem* AND HARAN, N.—(1955) *Brit. J. Cancer*, **9**, 453.
*Idem* AND SHUBIK, P.—(1947*a*) *Ibid.*, **1**, 379.—(1947*b*) *Ibid.*, **1**, 383.—(1949) *Ibid.*, **3** 109.
BOUTWELL, R. K., RUSCH, H. P. AND BOSCH, D.—(1955) *Proc. Amer. Ass. Cancer Res.*, **2**, 6.
FOULDS, L.—(1954) *Cancer Res.*, **14**, 327.
FRIEDEWALD, W. F. AND ROUS, P.—(1950) *J. exp. Med.*, **91**, 459.
GRAFFI, A., VLAMYNCH, E., HOFFMAN, F. AND SCHULTZ, I.—(1953) *Arch. Geschwulstforsch.*, **5**, 110.
HADDOW, A.—(1939) *Ann. Rep., Brit. Emp. Cancer Campgn.*, **16**, 304.
IRWIN, J. O.—(1949) *J. Hyg., Camb.*, **47**, 188.
*Idem* AND GOODMAN, N.—(1946) *Ibid.*, **44**, 362.
LAW, L. W.—(1941) *Amer. J. Path.*, **17**, 827.
ROE, F. J. C.—(1956*a*) *Brit. J. Cancer*, **10**, 61.—(1956*b*) *Ibid.*, **10**, 72.
*Idem* AND SALAMAN, M. H.—(1954) *Ibid.*, **8**, 666.—(1955) *Ibid.*, **9**, 177.
SALAMAN, M. H. AND GWYNN, R. H.—(1951) *Ibid.*, **5**, 252.
*Idem* AND ROE, F. J. C.—(1953) *Ibid.*, **7**, 472.—(1956) *Ibid*, **10**, 70.
SHUBIK, P.—(1950) *Cancer Res.*, **10**, 713.
*Idem*, BASERGA, R. AND RITCHIE, A. C.—(1953) *Brit. J. Cancer*, **7**, 342.
*Idem* AND RITCHIE, A. C.—(1953) *Cancer Res.*, **13**, 343.